# Toward a Mechanistic Framework for Intestinal Drug Permeability: Integrating *In Silico* Modeling with Biorelevant Assays

**DOI:** 10.1021/acsomega.5c09260

**Published:** 2026-02-26

**Authors:** Fábio J.N. Ferreira, Tallita Marques Machado, Fernanda Guilhon-Simplicio

**Affiliations:** † Faculty of Pharmaceutical Sciences, 67892 Federal University of Amazonas, Manaus, Amazonas 69080-900, Brazil; ‡ Center for Innovation in Artificial Intelligence for Health (CIIA − Saúde/UFMG), Belo Horizonte 31270-901, Brazil

## Abstract

Predicting oral bioavailability remains a central challenge in pharmaceutical sciences, primarily limited by the phenomenological nature of traditional predictive models that provide correlations without mechanistic insight. While molecular dynamics (MD) simulations provide detailed atomistic insights into drug-membrane interactions, they require rigorous experimental validation. Conversely, biorelevant assays—such as Caco-2 monolayers and everted gut sacs—supply essential biological end points but with limited mechanistic granularity. This review systematically evaluates the strengths and limitations of disparate approaches, from static quantitative structure–property relationship (QSAR) models to physics-based molecular simulations. We propose an integrated framework that synergistically combines the physical resolution of multiscale MD modeling with the biological relevance of hierarchical experimental validation. Using a representative molecule with a divergent pharmacokinetic profile—characterized by high predicted permeability yet substantial metabolic instability—as an exemplary case, we present a mechanistic workflow for resolving such discrepancies. This integrated approach transforms the validation process from a binary outcome into a diagnostic tool for mechanistic deconstruction, ultimately guiding the rational design of next-generation orally bioavailable therapeutics.

## Graphical abstract

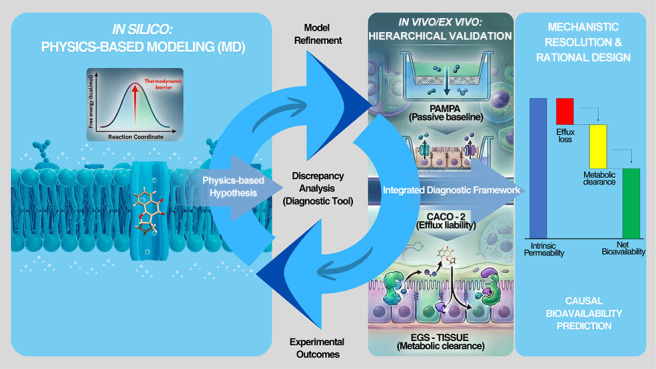

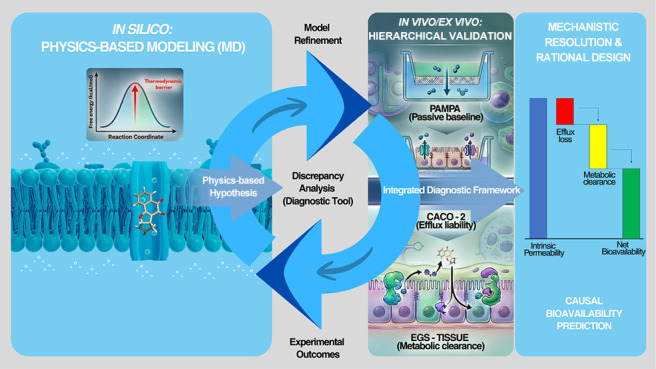

## 1 Introduction

### 1.1 From Phenomenological Classification to Mechanistic Understanding

The oral route remains the standard for drug administration due to high patient compliance and cost-effectiveness. However, achieving adequate oral bioavailability is a primary challenge in drug development and a significant factor in the attrition of drug candidates.^1^ The translocation of a molecule from the intestinal lumen to systemic circulation is restricted by biological and physicochemical barriers, notably intestinal permeability and first-pass metabolism.

The Biopharmaceutics Classification System (BCS)^2^ established a framework for categorizing drugs based on aqueous solubility and intestinal permeability. While the BCS is essential for regulatory guidance and formulation strategy—particularly in determining biowaiver eligibility—it is, by design, a phenomenological system. It categorizes compounds based on macroscopic outcomes but does not explain the molecular-level mechanisms underlying these properties.^3^ Consequently, the BCS cannot provide insights into the energetic costs of desolvation, the specific interactions between a ligand and the lipid bilayer, or the atomic-scale features that govern recognition by metabolic enzymes or efflux transporters.

Modern molecular pharmaceutics seeks to move beyond empirical categorization toward the elucidation of causal physical mechanisms.^4^ This mechanistic approach is necessary to transition drug development from empirical screening optimization to rational, predictive design. Understanding the correlation between molecular structure and pharmacokinetic profile requires tools that can probe translocation at the atomic scale, characterizing the energetic landscapes and dynamic trajectories of drug-membrane interactions.

### 1.2 The Role and Limits of Static Descriptors

To prioritize candidates in early stage discovery, the field has relied on computational filters and heuristics. Lipinski’s “Rule of Five” (Ro5)^5^ provides a widely used set of physicochemical boundaries (molecular weight, lipophilicity, and hydrogen bond counts) associated with oral absorption. Similarly, Quantitative Structure–Activity/Property Relationship (QSAR/QSPR) models utilize molecular descriptors to predict experimental end points, such as Caco-2 permeability (P_app_).^6^

Although effective for high-throughput screening, these models are inherently correlational.^7^ They identify structural features statistically associated with absorption but do not provide a mechanistic explanation for the observed behavior. Furthermore, these models typically treat the drug as a static entity. Membrane permeation, however, is a dynamic, multistep process involving sequential interactions with heterogeneous membrane environments. Static descriptors like LogP or Polar Surface Area (PSA) cannot fully capture the transient hydrogen bonding, conformational changes, or the specific energetic penalties associated with desolvation as a molecule traverses the lipid bilayer.^8^

Therefore, while useful for initial ranking, static models may fail to resolve cases where competing dynamic processes—such as passive diffusion, active transport, and metabolism—determine the final pharmacokinetic outcome.

### 1.3 Pharmacokinetic Challenges: The Case of CNFD

The limitations of static, correlational models are evident in the development of 6b,7-dihydro-5*H*-cyclopenta­[*b*]­naphtho­[2,1-*d*]­furan-5,6­(9a*H*)-dione (CNFD), a semisynthetic naphthoquinone with potential antineoplastic activity. Based on its physicochemical properties, CNFD meets conventional filters for oral absorption, leading to *in silico* predictions of high human intestinal absorption.^9^

However, this predicted permeability is offset by significant metabolic instability. *In vitro* studies using human liver microsomes indicate that CNFD is a substrate for cytochrome P450 enzymes, undergoing rapid metabolic clearance.^10^ This presents a significant pharmacokinetic challenge: a molecule predicted to cross the intestinal barrier efficiently may still exhibit low systemic bioavailability due to extensive presystemic metabolism in both the enterocytes and the liver. This divergence illustrates the limitations of relying on siloed descriptors; a model focused solely on passive permeation would classify CNFD favorably, omitting the metabolic liabilities that govern its *in vivo* performance.

Addressing these complexities requires an integrated investigative framework to address the following fundamental questions:

- How do high predicted intrinsic permeability and extensive first-pass metabolism (intestinal and hepatic) quantitatively impact net bioavailability?
- To what extent is low bioavailability driven by poor absorption versus rapid presystemic clearance?
- What are the specific molecular mechanisms governing the translocation of compounds like CNFD across the intestinal epithelium?

### 1.4 Scope of This Review

This review provides a systematic evaluation of current methodologies used to assess drug permeability and absorption. We first examine conventional *in vitro* and *ex vivo* experimental models, discussing their utility and inherent limitations in separating permeability from metabolism. We then explore the application of molecular dynamics (MD) simulations and physics-based computational modeling to quantify the thermodynamic and kinetic barriers of membrane translocation. Finally, we propose a synergistic framework that integrates these experimental and *in silico* approaches to provide a more accurate, mechanistically grounded prediction of oral bioavailability, using CNFD and other complex molecules as case studies for this integrated approach.

## 2 Experimental Models for Intestinal Permeability Assessment

### 2.1 Parallel Artificial Membrane Permeability Assay (PAMPA)

The Parallel Artificial Membrane Permeability Assay (PAMPA) provides a high-throughput, reductionist approach by eliminating biological complexity to isolate passive transcellular diffusion.^11,12^ This method utilizes an artificial phospholipid membrane supported on a filter, devoid of active transport mechanisms, metabolic enzymes, or tight junctions.

Due to its cost-effectiveness and scalability, PAMPA is particularly valuable in early stage screening for ranking compounds based on their intrinsic passive permeability.^13^ Furthermore, when used in conjunction with cellular models, PAMPA serves as a diagnostic tool for identifying transport mechanisms. A significant discrepancy where PAMPA permeability (representing passive diffusion) exceeds cellular permeability suggests the involvement of active efflux systems. Conversely, when cellular permeability surpasses that of PAMPA, carrier-mediated uptake or paracellular transport mechanisms are likely implicated.^14^

However, the simplicity of PAMPA constitutes its primary limitation. The absence of transporters and metabolic enzymes limits the assay’s capacity to capture the biologically mediated processes that frequently govern *in vivo* absorption kinetics.^15^

### 2.2 Caco-2 Cell Monolayers

The Caco-2 cell line, derived from a human colorectal adenocarcinoma, represents the standard *in vitro* model for intestinal permeability assessment, widely accepted by regulatory agencies such as the FDA and EMA for biopharmaceutical classification.^16–18^ Upon differentiation for approximately 21 days on semipermeable supports, these cells form a polarized monolayer characterized by apical microvilli and functional tight junctions that limit paracellular flux.^19,20^

The primary experimental output is the apparent permeability coefficient (P_app_), Bidirectional transport studies allow for the calculation of the efflux ratio (ER = P_app,B→A_/P_app,A→B_), where values exceeding 2.0 generally indicate the activity of efflux transporters, such as P-glycoprotein (P-gp).^21,22^

Despite its broad adoption, the P_app_ value is a composite end point that aggregates passive diffusion with active transport processes and potential system-specific artifacts.^23^ Two principal mechanistic limitations require consideration regarding the physiological relevance of this model:

#### 2.2.1 Transporter Expression Variability

While Caco-2 cells functionally express efflux transporters, the expression levels may not quantitatively reflect those found in the native human intestine. Studies indicate that while the overall transporter expression pattern in Caco-2 cells resembles that of the small intestine, significant quantitative differences exist for specific transporters such as OATP-B, BCRP, and MRP2 relative to human jejunal or ileal tissue.^24^ Furthermore, P-gp expression in Caco-2 cultures can vary depending on passage number and culture conditions, complicating the extrapolation of *in vitro* efflux ratios to *in vivo* absorption.

#### 2.2.2 Metabolic and Physiological Limitations

The metabolic capacity of standard Caco-2 cells is significantly lower than that of the human intestine. Notably, the expression of CYP3A4—the dominant intestinal drug-metabolizing enzyme—is negligible in conventional Caco-2 cultures, which limits the model’s utility for compounds undergoing extensive intestinal first-pass metabolism.^25^ Additionally, the monoculture lacks mucus-secreting goblet cells and a physiological mucus layer.^26^ The unstirred water layer (UWL) in static systems may also become rate-limiting for highly lipophilic compounds, potentially confounding the measurement of membrane translocation kinetics.^27,28^

Caco-2 provides a robust method for initial compound ranking and efflux assessment. However, for molecules where the pharmacokinetic profile is driven by a complex interaction between permeation and metabolism, Caco-2 assays provide a net transport value but may not fully elucidate the underlying molecular mechanisms.

### 2.3 Everted Intestinal Tissue Preparation

The everted gut sac (EGS) model offers increased physiological relevance by preserving native tissue architecture^29,30^ This *ex vivo* technique utilizes a freshly excised intestinal segment—typically from rat—inverted to expose the mucosal surface to the drug-containing buffer.

The EGS directly addresses principal Caco-2 deficiencies through: (a) intact three-dimensional tissue architecture including villi, crypts, and the native cellular population; (b) a functional apical mucus layer; and (c) complete metabolic competence with physiologically relevant Phase I (CYP3A4) and Phase II (UGT) enzyme activity.^31,32^ This enables a direct, simultaneous study of permeability and first-pass intestinal metabolism—quantifying the fraction of drug escaping the gut wall (F_g_)—a parameter Caco-2 cannot reliably determine.^33,34^

However, gains in physiological relevance incur significant costs. Tissue viability is limited to approximately 60–120 min, demanding routine monitoring via glucose transport confirmation or lactate dehydrogenase release.^35^ Interspecies variability introduces uncertainty when extrapolating rat tissue findings to human pharmacokinetics, as transporter isoforms (e.g., rat Mdr1a versus human P-gp) and enzyme specificities differ.^36^ Additionally, the static hydrodynamic environment does not replicate dynamic shear forces, meaning the UWL remains a significant diffusion barrier for highly permeable compounds.^37^

The EGS is not a high-throughput screening tool but a mechanistic instrument that deliberately trades stability and scalability for a brief window of physiological fidelity.

### 2.4 Next-Generation *In Vitro* Systems: Intestinal Organoids and Microphysiological Models

Intestinal organoids and microfluidic gut-on-chip devices represent advanced platforms addressing limitations of both Caco-2 and traditional *ex vivo* models.^38,39^ Human intestinal organoids, derived from adult stem cells, recapitulate the crypt-villus architecture and cellular diversity of native epithelium—including enterocytes, goblet cells, and enteroendocrine cells—while maintaining patient-specific genetic backgrounds.^40,41^ Recent advances demonstrate that organoid-derived monolayers achieve CYP3A4 activity comparable to or exceeding that of adult small intestine, with permeability correlating well with human fraction absorbed values (R^2^ = 0.88).^42,43^

Gut-on-chip systems extend this complexity by incorporating dynamic fluid flow, mechanical peristaltic motion, and capability for coculture with immune cells or microbiome components.^44,45^ These systems better mimic physiological conditions, with increased drug sensitivity observed compared to static Transwell models.^46^

However, technical complexity, limited standardization, and low throughput currently restrict organoid and chip applications to mechanistic studies of high-value compounds rather than routine screening.^47^ Nevertheless, their integration into hierarchical validation strategies represents a promising trajectory.

### 2.5 Limitations of Experimental Permeability Assessment

A comparative analysis of these experimental models (Table 1) reveals a unifying limitation: while each provides essential macroscopic end points, they treat the permeation process as an operational phenomenon without mechanistic granularity to describe the translocation process itself.^48^

**Table 1:** Comparative Assessment of Experimental Models for Intestinal Permeability Evaluation^a^

Model	Principle	Strengths	Limitations	Biological Relevance	Throughput
**PAMPA**	Artificial phospholipid membrane on filter support.	Rapid; cost-effective; isolates passive permeation.	No active transport; no metabolism; no cellular components.	Low (Pure physical partitioning)	Very High
**Caco-2**	Human adenocarcinoma cell monolayer forming polarized barrier.	Gold-standard; measures P_app_ and efflux ratio; regulatory acceptance.	P-gp variability; negligible CYP3A4 activity; lack of physiological mucus layer.	Moderate (Colonic origin; transporter expression variability)	High
**Everted Gut Sac**	Inverted intestinal segment preserving tissue architecture.	Intact architecture; full metabolic competence (CYP/Phase II); mucus layer present.	Limited viability (60–120 min); interspecies variability; labor-intensive.	High (Native tissue with enzymes and transporters)	Low
**Intestinal Organoids**	3D stem cell-derived structures recapitulating crypt-villus axis.	Human-derived; high cellular diversity; patient-specific potential.	Technical complexity; restricted apical access; standardization challenges.	Very High (Human origin; 3D architecture)	Low
**Gut-on-Chip**	Microfluidic device with human cells under dynamic flow.	Mimics physiological shear forces; coculture and real-time monitoring.	High operational cost; specialized equipment; limited validation data.	Very High (Dynamic microenvironment)	Low

a Throughput is classified relative to industrial screening demands. Biological relevance reflects the fidelity to human jejunal physiology.

The P_app_ value—whether from cell culture, artificial membrane, or tissue explant—is a composite outcome. Experimental models measure overall transport rates but lack the resolution to elucidate: (a) the preferred molecular orientation during membrane approach and translocation; or (b) the precise thermodynamic cost (free energy barrier) required for the molecule to partition into and cross the hydrophobic membrane interior.^49,50^

Consequently, this lack of molecular resolution creates interpretive uncertainty when interpreting pharmacokinetic data. A low experimental P_app_ could arise from mechanistically distinct scenarios: poor intrinsic diffusivity, significant transporter-mediated efflux, or rapid metabolic degradation.^51^ Experimental outcomes alone often cannot distinguish between these competing factors, making it difficult to determine whether a compound fails due to physical barriers (permeation) or biological barriers (efflux/metabolism).

Therefore, while experimental assays provide the essential benchmark for validation, they primarily quantify the net outcome rather than elucidating the underlying molecular process. To advance from a descriptive to a mechanistic understanding, computational approaches capable of resolving atomistic interactions—specifically molecular dynamics simulations—are required.^52,53^

## 3 Molecular Dynamics Simulations for Mechanistic Insight into Membrane Permeation

While experimental assays quantify the macroscopic outcomes of permeability, they provide limited insight into the underlying molecular events. To elucidate these mechanisms, methods capable of spatiotemporal resolution at the atomic scale are required. Molecular dynamics (MD) simulations address this need by numerically solving Newton’s equations of motion for the entire system—solute, solvated lipid bilayer, and ions. This approach generates dynamic, high-resolution trajectories of the permeation process, characterizing the specific interactions governing drug translocation.^54^ The utility of MD lies not merely in predicting permeability coefficients, but in providing the mechanistic data necessary to explain them.

It is important to distinguish between computational tools designed for high-throughput screening and those engineered for mechanistic investigation (Table 2). Static models, such as QSAR and Rule-of-Five heuristics, offer ultrahigh throughput and are essential for library filtering. However, these correlation-based methods cannot elucidate the physical origins of a permeability profile. Conversely, Enhanced Sampling MD simulations, despite lower throughput, provide the necessary thermodynamic resolution to deconstruct complex cases. Thus, the choice of methodology is dictated by the discovery stage: static models for broad filtering, and physics-based simulations for the deep mechanistic resolution of selected candidates.

**Table 2:** Hierarchy of *In Silico* Methodologies for Permeability Assessment, Differentiating between Statistical Screening Tools and Physics-Based Mechanistic Investigations

Methodology	Principle	Mechanistic Insight	Primary Application	Throughput
**QSAR/QSPR Models**	Statistical correlation between static molecular descriptors (e.g., LogP, PSA) and experimental end points.	Limited (Empirical correlations; lacks causal derivation).	High-throughput virtual screening; filtering large libraries (e.g., Ro5 compliance).	Ultra-High
**Unbiased Molecular Dynamics (MD)**	Simulation of atomic trajectories using Newton’s equations without artificial biasing forces.	Qualitative (Visualizes spontaneous interactions, orientation, and membrane perturbation)	Observing dynamic behavior and conformational changes at the lipid interface.	Low
**Enhanced Sampling MD (e.g., Umbrella Sampling)**	Biased simulation to force translocation and calculate free energy profiles (PMF, ΔG).	Quantitative (Calculates precise thermodynamic barriers and local diffusivity)	Rigorous mechanistic deconstruction of complex cases; inputs for the ISD model.	Very Low
**Integrated MD/ML& ML Potentials**	Machine Learning algorithms trained on physics-based MD data or quantum calculations (ML Force Fields).	**High** (Physics-informed; derived from first principle.	Accelerating free energy calculations and expanding prediction to broader chemical spaces.	Moderate/High

### 3.1 Physics-Based Descriptors of Permeation

MD simulations facilitate the decomposition of the aggregate permeability coefficient into its constituent thermodynamic and kinetic components.

#### 3.1.1 Potential of Mean Force (PMF) Profiles

The translocation of a drug across a membrane is a thermodynamic process. The Potential of Mean Force (PMF), denoted as ΔG­(z), quantifies the free energy of the system as a function of the drug’s position (z) along the membrane normal.^55^

Calculated using enhanced sampling techniques such as umbrella sampling or metadynamics, the PMF reveals mechanistic features inaccessible to standard experiments. The depth of energy wells at the interface quantifies the partitioning propensity, while the height of the central energy barrier ΔG_barrier_ represents the direct thermodynamic cost for the solute to cross the hydrophobic core.^56^ A lower ΔG_barrier_ correlates with higher intrinsic permeability. This provides a quantitative assessment of the thermodynamic hurdle for passive diffusion, isolating it from the contributions of active transport and metabolism.

#### 3.1.2 Diffusion Coefficients and the Inhomogeneous Solubility-Diffusion Model

Thermodynamics alone does not dictate the rate of transport; kinetics also play a critical role. A molecule may energetically favor a specific membrane region but diffuse through it slowly due to viscous drag or specific interactions. To capture this, MD simulations calculate the position-dependent diffusion coefficient, D­(z).

The D­(z) profile is integrated with the PMF using the Inhomogeneous Solubility-Diffusion (ISD) model.^49,57^ This model defines the intrinsic permeability coefficient (P) as inversely proportional to the total resistance across the membrane. This approach provides a rigorous calculation of (P) that is comparable to experimental P_app_ values, while simultaneously identifying kinetic traps—regions of low mobility that retard the overall transport process.

#### 3.1.3 Atomistic Interactions and Membrane Perturbation

Beyond energetics, MD simulations reveal the specific intermolecular interactions driving the observed landscape. Trajectory analysis allows for the visualization of:

- Orientation and Solvation: Determining the preferred orientation of the compound at the interface and quantifying the energetic penalty associated with desolvation.
- Intermolecular Bonding: Identifying transient hydrogen bonds formed between the solute’s polar groups and lipid headgroups (phosphate/glycerol) or water molecules.^58,59^

- Membrane Perturbation: Measuring the local disruption of lipid packing, bilayer thickness, or acyl chain order induced by the permeant.^60^

These atomistic details provide mechanistic hypotheses for rational drug design. For example, if a specific hydrogen bond is identified as the primary contributor to a high energy barrier, structural analogues can be designed to modulate this interaction.

### 3.2 Integration of Machine Learning with Molecular Dynamics

While MD provides high mechanistic resolution, its application to large compound libraries is limited by high computational costs. Machine learning (ML) addresses these computational constraints, not by replacing physics-based modeling, but by accelerating simulation throughput and extracting deeper insights from complex trajectories.

#### 3.2.1 Physics-Informed Predictive Models

Standard QSAR models typically rely on static topological descriptors. In contrast, an integrated approach utilizes dynamic, physics-based features derived from molecular dynamics as inputs for machine learning algorithms. For instance, calculated free energy barriers (ΔG_barrier_) and diffusion coefficients (D_avg_) serve as high-fidelity descriptors for training regression models. This integration is substantiated by recent computational studies: Dolz et al.^61^ and Karmakar et al.^62^ demonstrate the efficacy of utilizing energetic and diffusive barriers as input features for predictive modeling, while Leverant et al.^63^ and Tian et al.^64^ validate the methodology of deriving complex dynamic transport properties from advanced simulations to inform data-driven frameworks. This allows the model to learn the nonlinear relationships between energetic barriers and chemical structure, potentially improving generalizability to novel chemical spaces compared to correlations based solely on static properties.^65^ This creates a predictive tool that is inherently more robust and generalizable because it is grounded in the fundamental thermodynamics and kinetics of the permeation process, not just topological correlations.

#### 3.2.2 Unsupervised Trajectory Analysis

MD simulations generate extensive data sets containing atomic coordinates over millions of time steps. Manual analysis of such data is inefficient. Unsupervised ML techniques, such as dimensionality reduction and clustering algorithms, can automatically identify statistically significant conformational states and transition pathways adopted by the molecule during translocation.^66^ This automated analysis accelerates hypothesis generation by pinpointing the most relevant molecular events—such as specific lipid interactions or conformational distinct states—that govern the permeation mechanism.

#### 3.2.3 Acceleration via Machine Learning Potentials

An emerging development in computational chemistry is the use of ML to reduce the computational expense of free energy calculations. Machine Learning Potentials (MLPs) or Force Fields (MLFFs) are trained on high-accuracy quantum mechanical calculations to reproduce potential energy surfaces at a fraction of the cost of ab initio methods. By accelerating the evaluation of forces, these techniques enable the calculation of PMF profiles with quantum-level accuracy but at speeds approaching classical mechanics. This effectively increases the throughput of mechanistic investigations, making the rigorous assessment of multiple analogues feasible.

## 4 Synergistic Integration of *In Silico* and *In Vitro* Methods

The preceding analysis has critically dissected the individual strengths and molecular limitations of both experimental assays and computational simulations. While each pillar provides indispensable information, they remain incomplete in isolation. Experimental models offer biological reality without mechanistic transparency, while computational models provide physical transparency without inherent biological validation. The path forward for molecular pharmaceutics, therefore, lies not in choosing one approach over the other, but in their synergistic integration. This synergy is not merely advantageous; it is an imperative to elevate the science of drug permeability from a correlational practice to a truly causal and predictive discipline.

### 4.1 From Phenomenological Correlation to Mechanistic Causation

For decades, drug permeability assessment has been dominated by a correlational mindset. We measure a property *in vitro* (e.g., a P_app_ value) and seek a statistical correlation with an *in vivo* outcome (e.g., fraction absorbed).^16^ Similarly, traditional QSAR models correlate computational descriptors with experimental data. While useful, this approach is limited because correlation does not imply causation. A high P_app_ value correlates with good absorption, but it does not *explain* the molecular events—the thermodynamics of desolvation, the kinetics of membrane translocation, the interactions with lipid headgroups—that *cause* that high permeability. This is the critical distinction that an integrated framework addresses.

The rationale for integration is based on a systematic, bidirectional flow of information where each methodology remedies the deficiencies of the other.^67^

- ***In Vitro*** **Data Validates *In Silico*** **Physics:** MD simulations, for all their power, are based on approximations, from the force field parameters to the finite sampling of conformational space. The energetic landscape (PMF) and calculated intrinsic permeability (P) they produce are, fundamentally, theoretical predictions. The experimental P_app_ value, measured in a well-characterized system like a Caco-2 monolayer or an everted gut sac, serves as the essential “ground truth.” It provides the rigorous experimental benchmark against which the computational model must be validated.^67^ A strong correlation between the computationally predicted P and the experimentally measured P_app_ provides confidence that the underlying physics captured by the simulation is correct.
- ***In Silico*** **Physics Explains *In Vitro*** **Data:** Conversely, an experimental P_app_ value is an opaque, composite number. MD simulations provide the essential explanatory power to deconstruct this number into its constituent physical parts. They can reveal that a high P_app_ value is the result of a low desolvation penalty and rapid diffusion through the membrane core. They can explain *why* one analog has a higher permeability than another by pinpointing a specific hydrogen bond or a more favorable orientation within the bilayer.^68^ The simulation, therefore, provides the causal, molecular-level narrative for the experimental observation.

This integration transforms the scientific process; when predictions and experiments diverge, the discrepancy becomes a source of mechanistic insight. For instance, if MD predicts a high intrinsic permeability for molecule/drug, but the Caco-2 assay yields a low P_app_, the integrated framework strongly suggests that the difference is not due to flawed physics but to a biological process absent in the simplified MD model—namely, P-gp mediated efflux.^67,69,70^ By systematically comparing the outputs of pure diffusion models (MD), efflux-capable models (Caco-2), and metabolism-capable models (EGS), we can quantitatively dissect the relative contributions of each process to the final observed outcome.

Ultimately, this integration moves the field beyond simply building better predictive models to building better models of *understanding*. It establishes a direct line of sight from the quantum mechanical behavior of a molecule to its macroscopic pharmacokinetic properties, creating a powerful, validated framework for the rational design of new chemical entities with optimized oral bioavailability.

### 4.2 A Proposed Integrated Workflow for Investigating Molecular Permeability

To effectively integrate *in silico* predictions with *in vitro* data, a systematic and validated framework is required (Figure 1). This approach is not intended as a primary screening tool for large compound libraries—where high-throughput methods such as QSAR and PAMPA remain superior—but rather as a mechanistic diagnostic tool for investigating specific candidates, such as CNFD, that exhibit complex or discrepant pharmacokinetic profiles.

**Figure 1:**
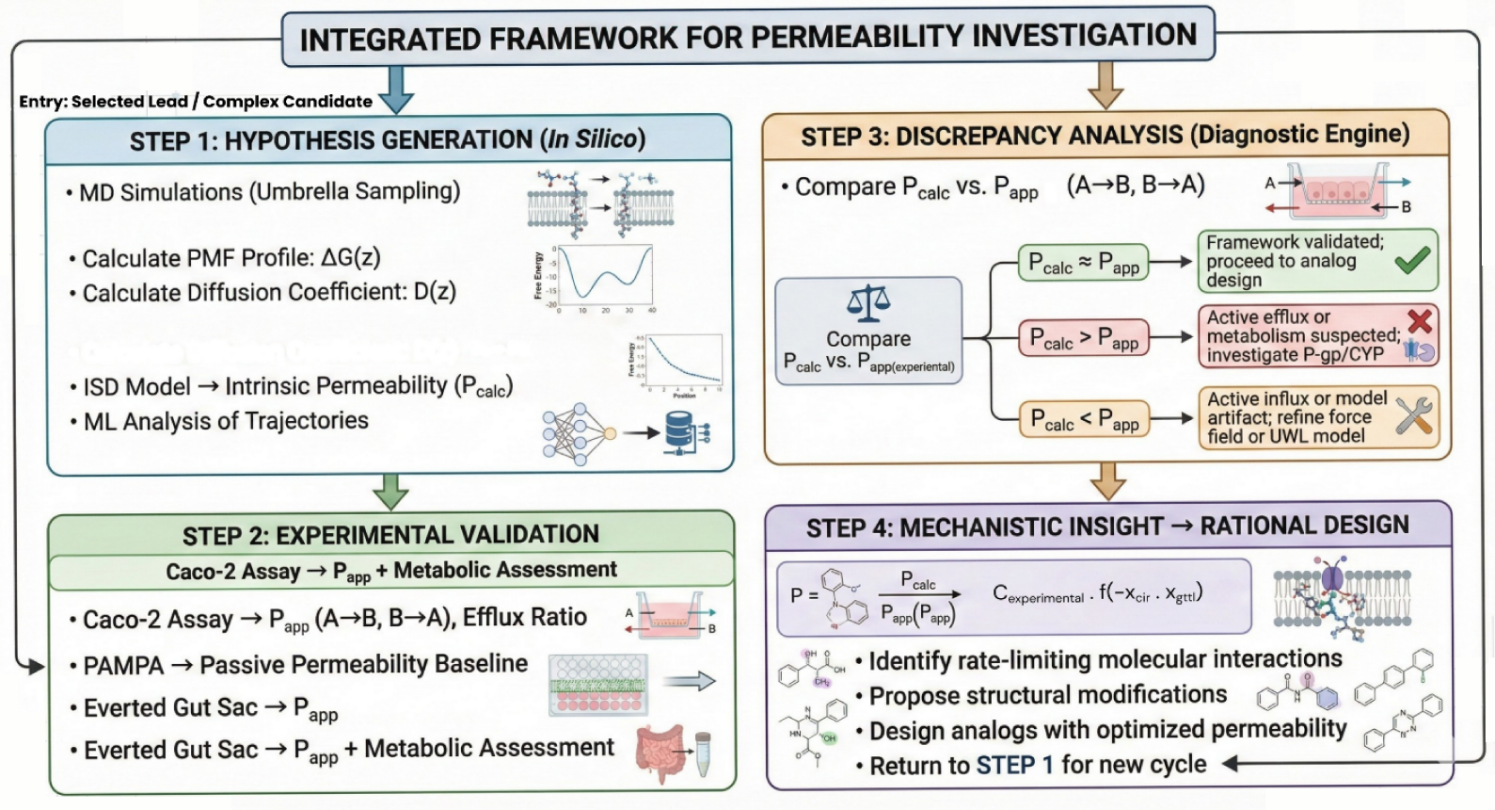
Integrated framework for the mechanistic investigation of selected drug candidates. The cycle is designed as a diagnostic engine for compounds exhibiting complex pharmacokinetic profiles, rather than a primary screening funnel. **Step 1:** The process initiates with physics-based hypothesis generation (MD) for a specific candidate. **Step 2:** Hierarchical experimental data is acquired to isolate transport mechanisms (PAMPA for passive, Caco-2 for efflux, EGS for metabolism). **Step 3:** Discrepancy analysis between *in silico* (P_calc_) and *in vitro* (P_app_) end points identifies biological confounders. **Step 4:** Mechanistic insights guide rational structural optimization.

For addressing complex pharmacokinetic divergences, we propose a workflow structured around four core principles:

#### 4.2.1 Physics-Based Hypothesis Generation

For a selected candidate of interest, the investigative cycle initiates with first-principles methods. Molecular dynamics simulations, by calculating the free energy landscape (PMF) of translocation, provide a clean, baseline prediction of a molecule’s intrinsic passive permeability. This yields a quantitative energetic hypothesis (e.g., ΔG_barrier_) that is fundamentally grounded in physics and deliberately disentangled from the complex and often confounding biological variables of active transport and metabolism.

#### 4.2.2 Hierarchical Experimental Validation

This physics-based hypothesis must then be rigorously benchmarked against a *hierarchy* of biorelevant assays. The data from a standardized *in vitro* model (such as a cell monolayer) can validate the simulation’s treatment of passive diffusion while providing a first quantitative measure of active transport processes. Subsequently, validation against a more physiologically complex ex vivo model (such as an intact intestinal tissue preparation) is essential to incorporate the crucial impact of gut wall metabolism. Viewing these experimental systems not as redundant but as distinct rungs on a ladder of biological complexity is central to this framework.

#### 4.2.3 Mechanistic Deconstruction through Discrepancy Analysis

The utility of this integrated workflow lies in the diagnostic interpretation of its discrepancies. These deviations are not failures but rich sources of mechanistic information. The quantitative gap between the predicted *in silico* intrinsic permeability and the measured *in vitro* apparent permeability can be leveraged to isolate and quantify the net impact of membrane transporters. Likewise, the difference between the *in vitro* and *ex vivo* outcomes can reveal the specific contribution of intestinal metabolism. This approach transforms the validation process from a simple pass/fail exercise into a powerful tool for mechanistic deconstruction.

#### 4.2.4 Mechanism-Guided Feedback and Rational Design

Ultimately, this cycle closes upon itself. The mechanistic insights gleaned from the discrepancy analysis—whether identifying a high transporter liability from the *in vitro* data or a significant metabolic liability from the *ex vivo* data—directly inform the next iteration of drug design. This ensures that the optimization of new analogues is not a process of trial and error, but a rational, data-driven effort guided by a validated, molecular-level understanding of the specific biological barriers that need to be overcome.

#### 4.2.5 Operational Considerations and Throughput

It is essential to distinguish the operational scope of this framework from routine screening. Unlike high-throughput campaigns, this integrated cycle functions as a low-throughput, high-content diagnostic strategy intended for high-value candidates.

Regarding the workflow timeline:

- Step 1 (*In Silico*): Enhanced sampling MD simulations, such as Umbrella Sampling, are computationally intensive. On modern GPU clusters, generating a converged free energy profile typically requires 3–7 days per compound. This positions the method as a downstream investigation tool for selected leads rather than an upstream filter.
- Step 2 (Experimental): To enable a rigorous discrepancy analysis (Step 3), data acquisition from multiple models is necessary. PAMPA establishes the passive diffusion baseline, Caco-2 identifies efflux liabilities, and the Everted Gut Sac elucidates metabolic clearance. Generating this comprehensive data set typically spans 2–3 weeks.

Consequently, the estimated throughput for one full iteration is approximately 1 month per lead candidate. While this time frame exceeds standard ADME profiling, it provides the mechanistic resolution necessary to deconstruct pharmacokinetic discrepancies that high-throughput assays alone cannot resolve.

The complete application of this framework to CNFD is currently under investigation in our laboratory. Therefore, the protocol presented serves as a methodological blueprint, substantiated by literature case studies. By isolating competing factors, this approach moves beyond the simple identification of discrepancies toward a mechanistic explanation of bioavailability, guiding systematic structural optimization.

### 4.3 Case Studies Supporting the Integrated Approach

While the systematic application of the proposed framework to CNFD is an ongoing investigation, the utility of integrating MD simulations with experimental assays is well-documented in recent literature. The following examples demonstrate how combining physics-based modeling with *in vitro* data allows for the mechanistic resolution of complex permeability profiles across diverse molecular classes.

#### 4.3.1 Discrepancy Analysis in Cyclic Peptides

Sugita et al. validated the diagnostic utility of comparing MD predictions with experimental data by calculating the membrane permeability of 156 cyclic peptides using replica-exchange umbrella sampling combined with the inhomogeneous solubility-diffusion (ISD) model.^69^ While the model showed significant correlation with PAMPA data for peptides with ALogP < 4, a systematic deviation was observed for highly lipophilic compounds. Crucially, this discrepancy was not a failure of the method but a diagnostic finding: it revealed that the ISD model alone did not account for the unstirred water layer (UWL) effects and membrane adsorption phenomena that dominate the transport of highly lipophilic species. This mirrors the “Discrepancy Analysis” step in our framework, where deviations identify missing physical or biological components.

#### 4.3.2 Mechanistic Explanation of Isomer Permeability: Withanolides

The challenge of distinguishing structurally similar compounds is exemplified by the study of withanolides by Wadhwa et al.^53^ Withaferin-A (Wi-A) and Withanone (Wi-N), two natural products differing primarily in hydroxyl positioning, exhibited differential cellular uptake profiles. PMF calculations elucidated the molecular basis of this difference: the terminal hydroxyl group in Wi-A enables favorable interactions with lipid phosphate headgroups, lowering the interfacial energy barrier compared to Wi-N. These computational insights provided a thermodynamic rationale for the experimentally observed differences in membrane accumulation and permeation, demonstrating the capacity of MD to resolve structural effects that static descriptors fail to capture.

#### 4.3.3 Conformation-Dependent Permeability in Macrocycles

Comeau et al. applied an integrated computational and experimental approach to 42 semipeptidic macrocycles.^71^ Their study highlighted the importance of conformational dynamics, as MD simulations in polar and apolar environments helped explain permeability differences between diastereomers (e.g., Nleu-5R vs Nleu-5S). The simulations revealed that intramolecular hydrogen bonding patterns, which vary between the isomers, significantly influence the desolvation penalty and membrane insertion cost. This study reinforces the premise that capturing dynamic conformational changes—rather than relying on static 3D structures—is essential for understanding the permeability of complex, flexible molecules.

### 4.4 Contextualization within the State-of-the-Art

The conceptual framework proposed herein represents the formalization of an emerging trend within molecular pharmaceutics. While leading research groups utilize specific elements of this integrated approach, the field currently lacks a standardized, diagnostic workflow that systematically leverages the discrepancies between methods to identify transport mechanisms.

Bridges between methods are being built. For instance, several studies^69,71–73^ have calculated free energy profiles (PMFs) for drug translocation, correlating ΔG_barrier_ with experimental P_app_ values. These foundational works confirm that MD simulations can capture the physics of passive translocation. However, these efforts often focus on validating the simulation against the experiment. The framework proposed here inverts this logic: it uses the validated simulation to interrogate the experiment, transforming the lack of correlation into mechanistic insight regarding metabolism or active transport.

Building on individual case studies, the systematic validation of large-scale MD simulations against experimental data sets represents a methodological development. The work by Sugita et al. (2021) establishes that computational methods can generate high-fidelity permeability data at scale, providing the essential *in silico* baseline required for the proposed discrepancy analysis. Furthermore, comprehensive reviews underscore the necessity of integrating computational, *in vitro*, and *in vivo* data to construct predictive absorption models, indicating a shift away from reliance on single-method approaches.

Concurrently, the integration of MD with machine learning addresses the computational constraints of physics-based methods. The underlying strategy involves utilizing computationally intensive MD simulations to generate high-fidelity physical data, which subsequently serves as “ground-truth” training sets for scalable ML models. Recent advances in large-scale AI for scientific discovery, such as those highlighted by Zheng et al. (2025),^74^ validate this approach. This confirms that coupling data-driven algorithms with physical simulations is an established strategy, reinforcing the relevance of the MD/ML component within the proposed framework.

These studies demonstrate that the fundamental components of the proposed workflow are currently available. While research exists bridging MD with *in vitro* data and integrating AI with simulation, the systematic articulation of these elements into a cohesive diagnostic framework remains limited. Specifically, a structured approach designed to investigate pharmacokinetic discrepancies—such as those observed with complex molecules—through iterative analysis is required. This review synthesizes these state-of-the-art methodologies into a unified strategy, formalized to address complex absorption profiles mechanistically.

## 5 Conclusion and Future Perspectives

The prediction of oral bioavailability remains a substantial challenge in pharmaceutical sciences. Complex pharmacokinetic profiles—exemplified by molecules such as CNFD—are governed by the competing rates of passive transcellular permeation, transporter-mediated active transport, and intestinal first-pass metabolism. Understanding these phenomena in isolation is insufficient; predictive modeling requires mechanistic insight into their quantitative integration.

Throughout this review, we have examined the molecular foundations of the field’s primary tools, including *in vitro* models (Caco-2 and PAMPA), *ex vivo* tissue preparations, and physics-based molecular dynamics simulations. While *in vitro* and *ex vivo* models yield necessary macroscopic end points, they remain phenomenological and limited in their ability to resolve underlying molecular mechanisms. Conversely, molecular dynamics simulations provide atomistic resolution but require experimental benchmarking to ensure biological relevance. Advancing predictive permeability models relies on the systematic integration of these approaches: MD simulations generate physics-based hypotheses regarding energetics and conformation, while hierarchical experimental assays provide the iterative feedback required to validate these predictions.

When computational predictions and experimental outcomes diverge, the discrepancy can serve as a diagnostic indicator rather than a model failure. A quantitative gap between calculated intrinsic permeability (P_calc_) and measured apparent permeability (P_app_) can isolate the contributions of efflux transporters, while differences between cell-based and tissue-based models elucidate the impact of intestinal metabolism. Thus, the proposed framework transforms validation into a mechanistic deconstruction, enabling the causal explanation of pharmacokinetic data.

Further developing this integrated framework will require advances in both computational and experimental methods. Computationally, future simulations must incorporate greater physiological complexity. Simple lipid bilayers can be limited approximations of the heterogeneous environment of intestinal epithelia; future models should account for membrane heterogeneity, including cholesterol and phase-separated microdomains. Furthermore, the integration of Hybrid Quantum Mechanics/Molecular Mechanics (QM/MM) methods may enable simulations of enzymatic reactions in more biologically relevant cellular environments, helping to bridge permeation and metabolic clearance within an integrated multiscale simulation.

Experimentally, the expansion of organoid-derived models and microphysiological systems is essential. These platforms recapitulate human tissue architecture and metabolic competence—features often absent in traditional Caco-2 monolayers. Standardization of these systems is required to enable their routine use in mechanism-driven assessments. Computationally, physics-informed machine learning (ML) offers a robust solution to current methodological bottlenecks; models trained on high-fidelity MD data can accelerate computationally intensive free energy calculations, addressing the computational constraints that currently limit the throughput of physics-based screening.

The complex pharmacokinetic profile of molecules such as CNFD can be viewed as a case study that can motivate methodological development. Applying the validated framework outlined herein can facilitate the quantitative analysis of the thermodynamic and kinetic balance governing passive permeability, active transport, and metabolism. By applying rigorous, mechanism-driven validation criteria, this approach supports efforts to move from empirical drug discovery toward mechanistically informed, multiscale predictive modeling.
